# pK_a_ of opioid ligands as a discriminating factor for side effects

**DOI:** 10.1038/s41598-019-55886-1

**Published:** 2019-12-18

**Authors:** Giovanna Del Vecchio, Dominika Labuz, Julia Temp, Viola Seitz, Michael Kloner, Roger Negrete, Antonio Rodriguez-Gaztelumendi, Marcus Weber, Halina Machelska, Christoph Stein

**Affiliations:** 1 Charité - Universitätsmedizin Berlin, Campus Benjamin Franklin, Department of Experimental Anesthesiology, Hindenburgdamm 30, 12203 Berlin, Germany; 2 Zuse Institute Berlin, Computational Molecular Design, Takustraße 7, 14195 Berlin, Germany; 3 Present Address: Department of Drug Discovery and In Vitro Pharmacology, Laboratorios Dr. Esteve, Parc Científic de Barcelona, Baldiri Reixac 4-12, Barcelona, Spain

**Keywords:** Behavioural methods, Peripheral nervous system

## Abstract

The non-selective activation of central and peripheral opioid receptors is a major shortcoming of currently available opioids. Targeting peripheral opioid receptors is a promising strategy to preclude side effects. Recently, we showed that fentanyl-derived μ-opioid receptor (MOR) agonists with reduced acid dissociation constants (pK_a_) due to introducing single fluorine atoms produced injury-restricted antinociception in rat models of inflammatory, postoperative and neuropathic pain. Here, we report that a new double-fluorinated compound (FF6) and fentanyl show similar pK_a_, MOR affinity and [^35^S]-GTPγS binding at low and physiological pH values. *In vivo*, FF6 produced antinociception in injured and non-injured tissue, and induced sedation and constipation. The comparison of several fentanyl derivatives revealed a correlation between pK_a_ values and pH-dependent MOR activation, antinociception and side effects. An opioid ligand’s pK_a_ value may be used as discriminating factor to design safer analgesics.

## Introduction

Opioids are the strongest drugs used for the treatment of pain, but serious problems have emerged due to their epidemic misuse and adverse effects (reviewed in^1^). Systemically applied conventional opioid agonists activate both central and peripheral opioid receptors (reviewed in^2–4^) and elicit major side effects such as sedation, respiratory depression, nausea, addiction, tolerance and constipation (reviewed in^1,5^). Targeting peripheral opioid receptors is a promising strategy to reduce adverse effects (reviewed in^6^). An increasing number of animal and clinical studies indicate that a large proportion of analgesia evoked by systemically administered opioids is mediated by such peripheral receptors^7–12^.

Many painful syndromes are associated with injury-induced tissue acidosis (reviewed in^6^) and low extracellular pH increases opioid agonist efficacy by altering the activation of opioid receptors and possibly G proteins^3,13–15^. In previous studies, we developed a novel artificial intelligence-based design of opioids lacking central or intestinal side effects by selectively targeting opioid receptors in the acidic environment of peripheral injured tissue. This approach aims at reducing a ligand’s pK_a_ by introducing electronegative fluorine atoms in order to preclude the protonation of its tertiary amine (an essential prerequisite for opioid receptor activation) at pH 7.4 (in brain and intestinal wall)^3,15,16^. In contrast to established wisdom that the pK_a_ of a drug affects pharmacokinetic characteristics such as absorption, distribution, metabolism and excretion (ADME)^17^, our novel concept is based on the different pharmacodynamics of opioid ligand-receptor interactions under physiological versus pathological conditions. However, the optimal pK_a_ to minimize side effects is not known to date. In this study, we tested the newly designed double-fluorinated compound *N*-{1-[2-(2,6-difluorophenyl)ethyl]piperidine-4yl}-*N*-phenylpropionamide (FF6), and examined whether successively decreasing an opioid ligand’s pK_a_ values correlates with the loss of central and intestinal side effects.

## Results

### Design, synthesis, and pK_a_ determination of FF6

**Figure 1:**
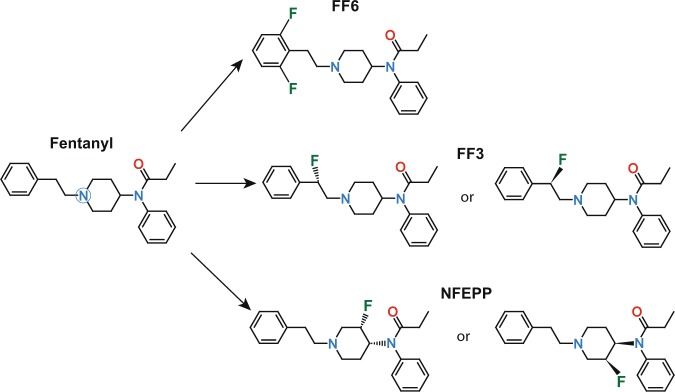
Chemical structures of fentanyl, N-{1-[2-(2,6-difluorphenyl)ethyl]piperidine-4-yl}-N-phenylpropionamide (FF6), (±)-*N*-[1-(2-fluoro-2-phenylethyl)piperidine-4-yl]-*N*-phenyl propionamide (FF3) and (±)-*N*-(3-fluoro-1-phenethylpiperidine-4-yl)-*N*-phenyl propionamide (NFEPP). The blue circle highlights the tertiary nitrogen atom subjected to pH-dependent protonation in whose vicinity electrons may be withdrawn to reduce the pK_a_ value. The respective isomers of FF3 and NFEPP are shown. Sites of fluorination are indicated as F.

Conventional fentanyl has an experimentally determined pK_a_ value of 8.44 ± 0.05^18^. To introduce electronegative moieties and facilitate chemical synthesis, two hydrogens were replaced by two fluorine atoms at the phenyl ring in the fentanyl structure (Fig. 1). Accordingly, FF6 was synthesized by a contractor (ASCA GmbH Berlin, Germany) and its pK_a_ was experimentally measured as 7.94 ± 0.01 by another contractor (Sirius Analytical Ltd., Forest Row, UK). This compound was compared to fentanyl and two previously described derivatives (±)-*N*-[1-(2-fluoro-2-phenylethyl)piperidine-4-yl]-*N*-phenyl propionamide (FF3) and (±)-*N*-(3-fluoro-1-phenethylpiperidine-4-yl)-*N*-phenyl propionamide (NFEPP) (Fig. 1).

### FF6 binds to and activates μ-opioid receptors (MOR) at both low and physiological pH

**Figure 2:**
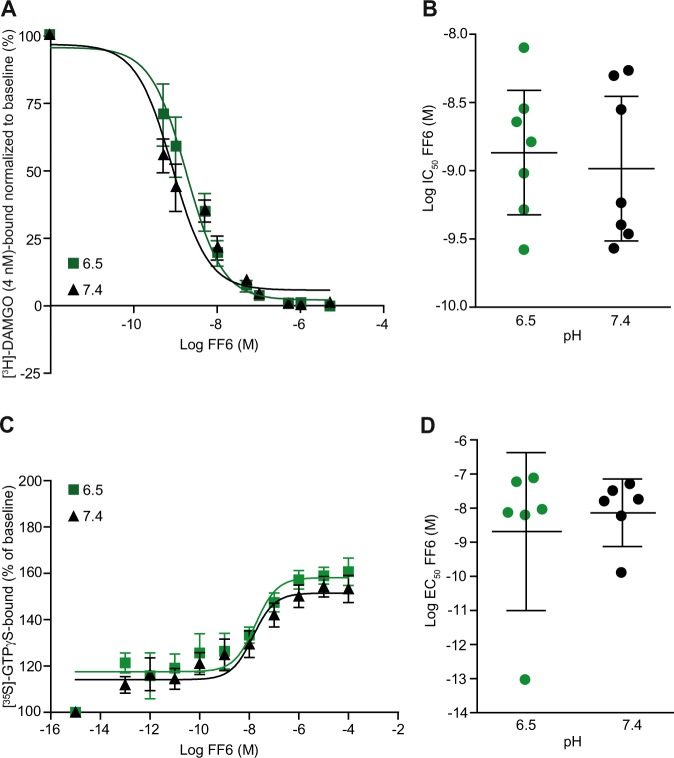
Binding and activation of MOR. (**A**) Displacement of bound [³H]-DAMGO (4 nM) by FF6 at pH 6.5 and 7.4. (**B**) IC_50_ calculated from (**A**). P > 0.05, unpaired *t*-test (n = 6–7). (**C**) [^35^S]-GTPγS binding induced by FF6 at pH 6.5 and 7.4. [^35^S]-GTPγS binding is expressed as percent increase in [^35^S]-GTPγS binding relative to binding in unstimulated samples (n = 6). (**D**) EC_50_ of FF6 from (**C**). P > 0.05, unpaired *t*-test. Data are presen*t*ed as mean ± SEM (A, C) and as mean ± 95% confidence intervals (B, D) (n = 6).

In binding experiments on membrane preparations of MOR-transfected human embryonic kidney 293 (HEK 293) cells, FF6 showed similar potency (IC_50_) to displace the radioactively-labeled standard MOR ligand [^3^H]-[D-Ala2,N-Me-Phe4,Gly5-ol]-enkephalin (DAMGO) (4 nM) at pH 6.5 and physiological pH 7.4 (Fig. 2A,B and Table 1). In the [^35^S]-GTPγS binding assay, both the maximum effects and the EC_50_ values of FF6 were similar at pH 6.5 and at pH 7.4 (Fig. 2C,D and Table 1).

**Table 1:** Comparison of *in vitro* effects of compounds with different pK_a_ values.

Drug	Calculated pK_a_	Experimentally obtained pK_a_	MOR binding (IC_50_ nM) in HEK 293 cells		Maximum [^35^S]-GTPγS binding at 100 μM agonist (% of baseline)		EC_50_ of [^35^S]-GTPγS binding (nM)	
			pH 6.5	pH 7.4	pH 6.5	pH 7.4	pH 6.5	pH 7.4
Fentanyl	9.11^3^	8.44 ± 0.05^18^	6.9 ± 1.1^15^	4.8 ± 0.7^15^	157.7 ± 9.1	144.1 ± 2.9	125.5 ± 20.6^3^	96.1 ± 16.6^3^
FF6	n/a	7.94 ± 0.01	2.3 CI: −0.1 to 4.6	2.0 CI: −0.002 to 4.1	157.3 ± 3.9	151.3 ± 4.4	22.8 CI: −6.8 to 52.4	17.9 CI: 1 to 34.8
FF3	6.01^3^	7.22 ± 0.01^3^	7.1 ± 1.4^3^	48.3 ± 6.4^***^^3^	129.9 ± 11.6	164.9 ± 21.4	47.2 ± 24.2^3^	231.4 ± 47.9^**^^3^
NFEPP	6.83^15^	6.82 ± 0.06^15^	18.3 ± 3.3^15^	81.9 ± 22.1^*^^15^	149.4 ± 10.6	137.7 ± 2.4	131.4 ± 29.8	337.7 ± 166.1

Data are represented as mean ± SEM (for normally distributed data) or mean with 95% confidence intervals (for non-normally distributed data). N.S., not significant P > 0.05, ^*^P < 0.05; ^**^P < 0.01; ^***^P < 0.0001; Mann-Whitney or unpaired *t-*test (n = 6–8); n/a: n.

### FF6 produces antinociception in healthy and injured tissue

**Figure 3:**
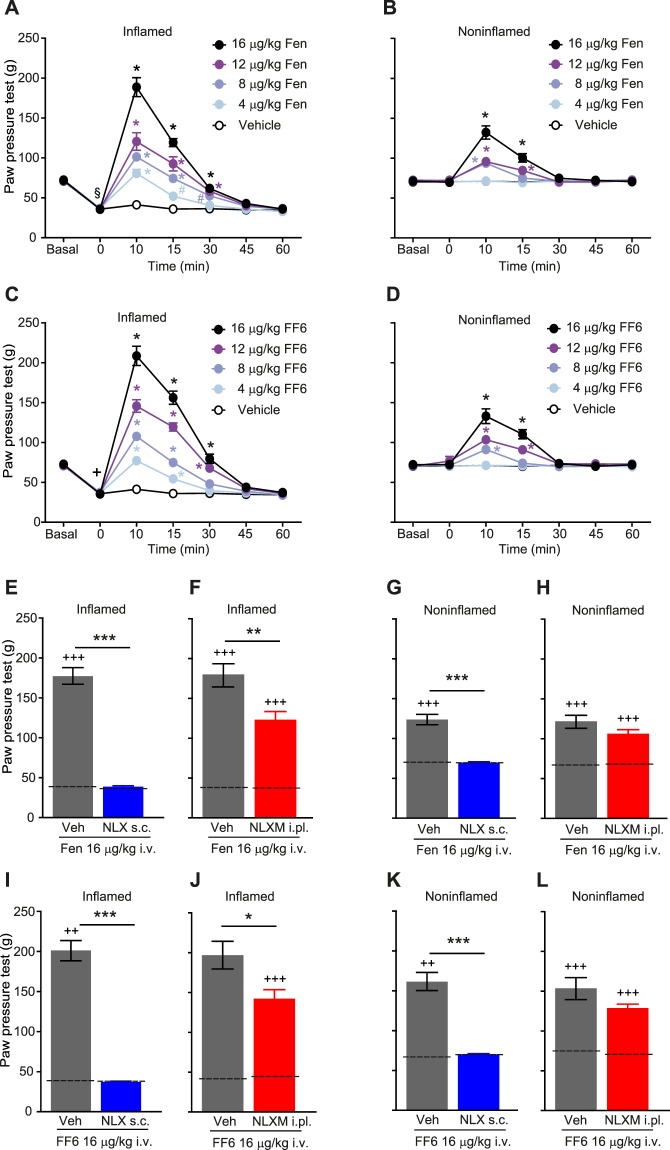
Antinociceptive effect of systemic FF6 in the unilateral CFA-induced hindpaw inflammation. (**A**–**D**) Elevation of PPT after intravenous (i.v.) injection of fentanyl (Fen) (**A**,**B**) and FF6 (**C**,**D**) in inflamed (**A**,**C**) and noninflamed (**B**,**D**) hindpaws, assessed before (Basal) and 4 days after i.pl. CFA application (0) at 10 to 60 min after injection of fentanyl or FF6. ^§^P < 0.01, ^+^P < 0.001 vs^.^ corresponding baseline threshold (Basal) before CFA injections, paired *t*-test or Wilcoxon tes*t*; ^#^P < 0.05, ^*^P < 0.001 vs. vehicle, two-way RM ANOVA and Bonferroni test (n = 8–10). (**E**–**L**) Effects of antagonists on PPT elevations produced 10 min after i.v. injection of fentanyl (**E**–**H**) or FF6 (**I**–**L**) (each at 16 µg/kg). Naloxone hydrochloride (NLX, 2 mg/kg) or vehicle (Veh) were injected subcutaneously (s.c.) (**E,G,I,K**). Naloxone methiodide (NLXM, 50 µg) or vehicle (Veh) were injected intraplantarly (i.pl.) into both hindpaws (**F**,**H**,**J**,**L**). ^*^P < 0.05, ^**^P < 0.01, ^***^P < 0.001 NLXM or NLX + Fen or FF6 vs. Veh + Fen or FF6, unpaired *t-*test; ^++^P < 0.01, ^+++^P < 0.0001 vs. corresponding baseline thresholds (dashed lines) evaluated 4 days after CFA, but before any injections; paired *t*-test or Wilcoxon test (n = 10 animals per condition for all except vehicle in J and L (n = 8)). Da*t*a are presented as mean ± SEM.

To assess antinociceptive efficacy, we used a clinically relevant rat model of pain, unilateral complete Freund’s adjuvant (CFA)-induced hindpaw inflammation^19^. Four days following intraplantar (i.pl.) CFA injection, rats developed mechanical hyperalgesia indicated by reduced paw pressure thresholds (PPT) in ipsilateral compared to contralateral paws, and to thresholds before injury (Fig. 3). Intravenous (i.v.) fentanyl (Fig. 3A,B) and FF6 (Fig. 3C,D) (4–16 μg/kg) produced dose-dependent antinociception manifested by increased PPT at 10–30 min after injection. These effects occurred both in inflamed (Fig. 3A,C) and contralateral, noninflamed paws (Fig. 3B,D). To examine the contribution of central vs. peripheral opioid receptors, we used subcutaneous (s.c.) administration of naloxone hydrochloride (NLX) and bilateral intraplantar (i.pl.) injection of naloxone methiodide (NLXM). These opioid receptor antagonists do^20^ or do not cross the blood-brain barrier^21^, respectively. The antinociceptive effects produced by fentanyl and FF6 (each at 16 μg/kg, i.v.) in inflamed paws were completely suppressed to the baseline thresholds before injections by NLX (2 mg/kg) (Fig. 3E,I). In contrast, the antinociception induced by both agonists was only partially abolished by NLXM (50 μg), as manifested by significantly different effects compared to baseline thresholds (Fig. 3F,J). The effects evoked by both agonists in contralateral, noninflamed paws were fully reversed by NLX to the baseline thresholds (Fig. 3G,K), but were not altered by NLXM, as demonstrated by the lack of significant differences compared with animals treated with both agonists and vehicle (Fig. 3H,L).

### FF6 induces central and intestinal side effects

**Figure 4:**
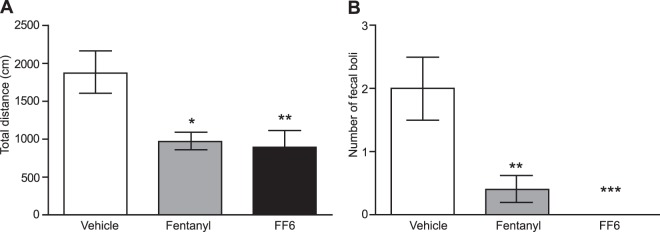
Central and intestinal side effects induced by systemic fentanyl and FF6. (**A**) Effects of subcutaneous (s.c.) fentanyl and FF6 (each at 30 µg/kg) on locomotion, expressed as the total distance (in cm) travelled during 30 min after drug injection. ^*^P < 0.05, ^**^P < 0.01 vs^.^ vehicle, one-way ANOVA and Dunnett’s test. (**B**) Effects of fentanyl and FF6 on constipation presented as the number of defecations during 1 h after s.c. fentanyl or FF6 injection. ^**^P < 0.01, ^***^P < 0.001 vs. vehicle, Kruskal-Wallis ANOVA and Dunn’s test. Data are presented as mean ± SEM (n = 10 animals per condition).

Next, we examined typical opioid side effects mediated centrally (sedation) or intestinally (constipation), as determined by locomotor activity and defecation, respectively. Both fentanyl and FF6 (each at 30 µg/kg, s.c.) decreased locomotor activity, measured as the distance traveled within 30 min after drug injections (Fig. 4A), and reduced defecation (Fig. 4B).

### pK_a_ values correlate with side effects

**Figure 5:**
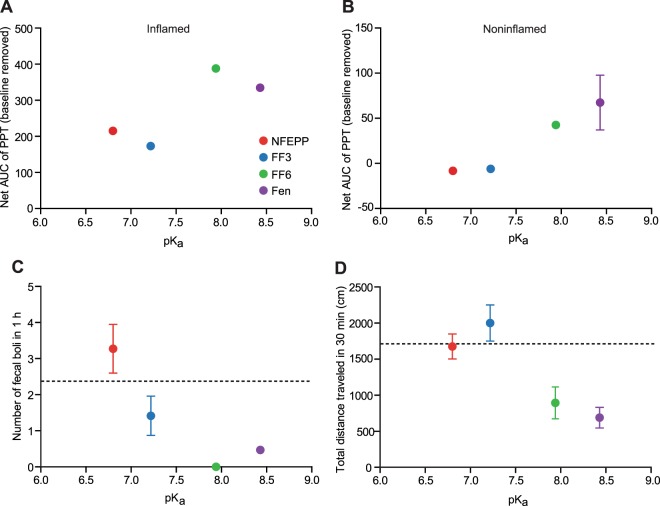
Correlation between pK_a_ values of compounds with antinociception and side effects. (**A,B**) Antinociceptive effects as net AUC_(4–12 μg/kg)_ of PPT (negative values result from subtraction of pre-CFA baseline PPT) at 15 min after i.v. injection of NFEPP, FF3, FF6 and Fen in inflamed (**A**) and contralateral, noninflamed (**B**) paws. AUC values were derived from curves generated by use of n = 9 (NFEPP and FF3 from^3,15^), n = 8–10 (FF6, see also Fig. 3) and n = 19 (Fen) animals. (**C**) Constipation, as assessed by number of fecal boli 1 h after s.c. injection of agonists (30 μg/kg) in relation to the pK_a_ of the substance. Maximum and minimum numbers (with 95% confidence intervals) of boli in controls (vehicle-treated) are shown by the dashed line. Fen (n = 30) and controls (n = 34) (always included in all experiments) are averaged across all experiments; NFEPP (n = 11); FF3 (n = 12); FF6 (n = 10). (**D**) Locomotor activity as assessed by the total distance travelled during 30 min after s.c. injection of agonists (30 μg/kg) in relation to the pK_a_ of the substance. Maximum and minimum travelled distances (with 95% CI) in controls (vehicle-treated) are shown by the dashed line. Fen (n = 31) and controls (n = 34) (always included in all experiments) are averaged across all experiments; NFEPP (n = 10); FF3 (n = 12); FF6 (n = 10). Graphs show means ± SEM (where available).

Finally, we gathered some *in vitro* and *in vivo* data from our previous studies^3,15,18^ to enable the direct comparison of the effects of all four compounds in physiological (pH 7.4) and acidic environments. FF6 (pK_a_ = 7.94) and fentanyl (pK_a_ = 8.44) showed similar MOR affinity and [^35^S]-GTPγS binding at both pH 6.5 and 7.4 (Table 1). FF3 (pK_a_ = 7.22) showed significantly enhanced potency to displace [^3^H]-DAMGO binding (i.e. increased MOR affinity) at low pH (Table 1). In the [^35^S]-GTPγS assay, both the maximum effect and the EC_50_ of FF3 were lower at pH 6.5 than at pH 7.4 (Table 1). NFEPP (pK_a_ = 6.82) displayed significantly enhanced MOR binding and showed a tendency to increase G-protein activation at pH 6.5 compared to pH 7.4 (Table 1). *In vivo*, FF6 and fentanyl (the ligands with high pK_a_) produced significantly elevated PPT over baselines (antinociception) both in inflamed and noninflamed paws (Fig. 3A–D; elevated AUC in Fig. 5A,B). In contrast, NFEPP and FF3 (the ligands with low pK_a_) did not affect noninflamed paws (AUC close to zero; Fig. 5B) and evoked antinociception only in inflamed paws (Fig. 5A) (all substances at 4–12 µg/kg, i.v.). Sedation and constipation were observed only after administration of the ligands with high (FF6, fentanyl) but not with low pK_a_ values (FF3, NFEPP) (all at 30 μg/kg, s.c.; Fig. 5C,D).

## Discussion

The activation of opioid receptors in peripheral inflamed (acidic) tissue is a promising strategy to reduce injury-induced pain and avoid central and intestinal side effects (reviewed in^6^). Conventional opioid agonists have pK_a_ values above 7.5 (reviewed in^3^). Therefore, their protonation and the subsequent activation of opioid receptors occur at both physiological and low pH^3,15^. Using computational simulations, we recently demonstrated that replacing a single hydrogen by a fluorine atom within a distance of two carbon bonds from the tertiary amine in the fentanyl molecule decreased the pK_a_ values of two derivatives (NFEPP, FF3) and promoted their selective protonation in inflamed tissue^3,15,16^. This lead to enhanced potency of MOR ligands at low pH *in vitro*, a finding that was confirmed by Dockendorff and colleagues^22^. In the current study, we examined a new compound created by replacing two hydrogens with two fluorine atoms at the phenylethyl ring (FF6).

The comparison of the four ligands revealed that the pK_a_ of FF6 (7.94) was higher than physiological pH and closer to the pK_a_ of fentanyl (8.44) than to the pK_a_ of FF3 (7.22) or NFEPP (6.82). Apparently, the newly introduced fluorine atoms were not able to sufficiently reduce pK_a_. This might be due to the already high electronegativity of the phenyl ring itself, or to the larger distance (four carbon bonds) between the fluorine atoms and the tertiary amine in FF6 than in NFEPP or FF3 (Fig. 1).

Consistent with their high pK_a_ values, FF6 and the standard MOR opioid agonist fentanyl induced comparable MOR binding and G-protein activation at both physiological and low pH, which is likely due to their similar protonation status under all pH conditions. In contrast, FF3 (pK_a_ = 7.22) and NFEPP (pK_a_ = 6.82) showed enhanced opioid binding and G-protein activation under acidic conditions, indicating that an increased proton concentration improved the interaction between ligands and opioid receptors.

When analyzing *in vivo* effects in correlation to pK_a_ values, we found that the ligands with high pK_a_ values (FF6, fentanyl) produced antinociception in both inflamed and in contralateral, non-inflamed paws, whereas the compounds with low pK_a_ (FF3, NFEPP) were inactive in noninflamed tissue. To discriminate between central and peripheral sites of action, we used s.c. NLX and i.pl. NLXM. The applied sites, doses and times of injection were based on previous studies that had shown that, at those modes of administration, NLX blocks both central and peripheral opioid receptors, whereas NLXM blocks only peripheral opioid receptors^15,23,24^. While systemic NLX abolished the effects of FF6 and fentanyl bilaterally, locally administered NLXM partially reduced only the effects in inflamed paws. In addition, both FF6 and fentanyl induced sedation and constipation. Together, these data indicate that FF6 and fentanyl activate both central (NLX-accessible) and peripheral (NLX- and NLXM-accessible) opioid receptors. These findings are consistent with our *in vitro* data and with the notion that both FF6 and fentanyl are protonated and capable of activating MOR at physiological (in brain or intestinal wall) as well as low pH (at the site of peripheral inflammation). Because we found no significant advantage of FF6 over conventional fentanyl, we did not further explore respiratory effects or addiction potential.

These results support our hypothesis that ligands with pK_a_ values close to the pH of inflamed/injured tissue selectively activate peripheral opioid receptors. The lack of pK_a_ reduction and the resulting absence of pH- and injury-specific action of FF6 confirmed this hypothesis. When comparing the present results with our previous *in vivo* studies on FF33 and NFEPP^15^, it appears that progressively decreasing pK_a_ values correlate with reduced sedation and constipation (Fig. 5C,D). Importantly, these experiments were conducted over a period of only two years under identical conditions in our laboratory by the same investigators. Although the results are reported in separate publications, we do not consider this a comparison of new with historical data or a limitation of the analysis. Of note, our approach does not exploit drug distribution, e.g. the entry into the brain or intestinal wall. In contrast to the known influence of the pK_a_ on a drug’s pharmacokinetic characteristics such as absorption or distribution^17^, our concept is based on the different pharmacodynamics of opioid ligand-receptor interactions under physiological versus pathological conditions. The chemical structures of our fluorinated derivatives are very close to fentanyl, a highly lipophilic molecule. Therefore, we expect the tissue distribution of NFEPP, FF3 and FF6 to be similar to fentanyl. As systemically administered fentanyl is known to rapidly enter the brain^25^, we assume that all substances are able to enter central and peripheral compartments. This will have to be verified in future investigations. However, as we have shown, the likelihood of producing adverse side effects by activating off-target opioid receptors at normal pH values (in brain, intestinal wall or other non-injured tissue) declines with decreasing pK_a_ values of agonists. Therefore, an opioid ligand’s pK_a_ value might be used as discriminating factor in the design of safer analgesics.

## Methods

### Chemicals/Drugs

Fentanyl citrate, NLX, NLXM, guanosine 5′-[γ-thio]triphosphate tetralithium salt (cold GTPγS) and guanosine 5′-diphosphate sodium salt (GDP) were purchased from Sigma-Aldrich (Taufkirchen, Germany). [^3^H]-DAMGO and [^35^S]-GTPγS were purchased from Perkin Elmer (Rodgau-Jügesheim, Germany). Isoflurane was purchased from AbbVie (Ludwigshafen, Germany), and CFA was purchased from Calbiochem (La Jolla, CA, USA).

FF6 (base) was synthesized by a contractor (ASCA GmbH, Berlin, Germany) (Fig. 1). The experimental measurement of pK_a_ was performed by a contractor (Sirius Analytical Ltd., Forest Row, UK). For *in vitro* experiments, fentanyl and FF6 were initially dissolved in water and dimethyl-sulfoxide (DMSO), respectively, and diluted in assay buffer to final concentrations. For *in vivo* experiments, FF6 was dissolved in DMSO and diluted with 0.9% NaCl to obtain the final concentrations. The maximum DMSO concentration was 4.2% for s.c., and 0.5% for i.v. injections. Fentanyl, NLX and NLXM were dissolved in water and diluted with 0.9% NaCl. Control groups were treated with vehicle (DMSO or NaCl, respectively). In the previously described fentanyl derivatives, fluorination of the ethylidene bridge yielded FF3 (experimental pK_a_ = 7.22)^3^, and fluorination of the piperidine ring lead to the compound NFEPP (experimental pK_a_ = 6.82)^15^ (Fig. 1 and Table 1).

### Cell cultures

HEK 293 cells (wild type or stably expressing rat MOR) were maintained in DMEM media (Sigma-Aldrich) supplemented with 10% fetal bovine serum and 1% penicillin/streptomycin in the absence or presence of 0.1 mg/ml geneticin (Biochrom AG, Berlin, Germany), respectively, in 5% CO_2_ at 37 °C. Depending on their density, cells were passaged 1:3–1:10 every second to third day from P8 to P28^26^.

### Radioligand binding assays

MOR-expressing HEK 293 cells were cultured in flasks with a growth area of 175 cm². Cells were washed twice with ice-cold assay buffer (Trizma® Preset Crystals, 50 mM, pH 7.4) (Sigma-Aldrich), then harvested from the culture flask in 10 ml ice-cold assay buffer, homogenized and centrifuged twice at 42,000 *g* for 20 min at 4 °C as described previously^24,27,28^. Protein concentration was determined according to the Bradford method^29^. The half-maximal inhibitory concentration (IC_50_) of FF6 required to displace 4 nM of the standard MOR ligand [^3^H]-DAMGO was determined at pH values 6.5 and 7.4. A protein amount of 100 µg was incubated with 4 nM [^3^H]-DAMGO (50 Ci/mmol) and FF6 dissolved in 50 mM assay buffer at pH 6.5 or 7.4 for 90 min at room temperature. Nonspecific binding was determined by the addition of 10 µM NLX^24^. Filters were soaked in 0.1% polyethyleneimine solution before use. Bound and free ligands were separated by rapid filtration under vacuum through Whatman GF/B glass fiber filters. Bound radioactivity was determined by liquid scintillation spectrophotometry at 69% counting efficiency for [^3^H] after overnight extraction of the filters in scintillation fluid.

For [^35^S]-GTPγS-binding experiments, membranes were prepared as described above. After determination of protein concentration, membranes were centrifuged as described above and resuspended in [^35^S]-GTPγS-binding assay buffer (100 mM NaCl, 50 mM Tris Base, 5 mM MgCl_2_, 0.1 mM EGTA, 0.2% bovine serum albumin, 10 mM dithiotreitol and 0.03 mM GDP) adjusted to pH 7.4 or 6.5^30^. A protein amount of 50 μg was incubated with 0.05 nM [^35^S]-GTPγS and varying concentrations of fentanyl derivatives at the respective pH for 2 h at 30 °C to determine dose response curves and EC_50_ values. Whatman GF/B glass fiber filters were soaked in water before use. Bound and free [^35^S]-GTPγS was separated *via* rapid filtration as described above. Nonspecific binding was determined by the addition of 10 μM cold GTPγS. Basal [^35^S]-GTPγS-binding was measured in the absence of opioid ligand and cold GTPγS.

### Animals

Experiments were performed in male Wistar rats (200–300 g, 6–7 weeks old, Janvier Laboratories, France) and approved by the State animal care committee (Landesamt für Gesundheit und Soziales, Berlin). All procedures were conducted in accordance with the ARRIVE guidelines^31^ and with the ethical guidelines of the International Association for the Study of Pain. Animals were randomly assigned to treatment or control groups for behavioral experiments. The experimenters were blinded to the doses and drug treatments. Rats were kept on a 12 h dark-light cycle in groups of 2–3 in cages lined with ground corncob bedding with free access to food and water *ad libitum*, and at constant room temperature (22–24 °C) and humidity (60–65%). Before nociceptive testing, handling was performed once per day for 4 days for 1–2 min each day. For assessment of locomotor activity, animals were habituated to the test cages one day before the experiment for 15 min. Statistical power calculations were performed to obtain the minimal number of animals for the experiments. After termination of the experiments, rats were killed by an overdose of isoflurane.

### Induction of hindpaw inflammation

Rats received an i.pl. injection of CFA (150 µl, 0.1% *Mycobacterium butyricum*) into the right hindpaw under brief isoflurane anesthesia^19^. Nociceptive testing was performed before and 4 days after CFA injection.

### Injections and experimental protocols

Brief isoflurane anesthesia was applied for i.v. (200 μl) injections. Nociceptive tests were performed in separate groups of animals, before and 5–60 min after drug injections. NLX was injected s.c. immediately before i.v. injection of agonists, similar to our previous study^23^. NLXM was injected i.pl. into both hindpaws immediately before i.v. injection of agonists, and pain thresholds were measured 10 min thereafter as described previously^24^. To avoid interference of general anesthesia with locomotor activity and to allow comparison with our previous studies^3,15^, subcutaneous (s.c.; 200 μl) injections without anesthesia were used for assessment of the other behavioral parameters. In addition, previous studies showed that ratios of peak plasma concentrations after i.v. versus s.c. administration of opioids were similar, and that these concentrations correlated well with antinociceptive and side effects^32–35^. All dosages were determined in pilot experiments.

### Mechanical hyperalgesia (Randall-Selitto test)

Rats were gently held under paper wadding and incremental pressure was applied via a wedge-shaped, blunt piston onto the dorsal surface of the hindpaws using an automated gauge (Ugo Basile, Comerio, Italy). The paw pressure threshold (PPT) necessary to induce paw withdrawal was determined by averaging three consecutive trials separated by 15 s intervals. The cut-off was set at 250 g to avoid tissue damage. The sequence of paws was alternated between animals to preclude order effects.

### Locomotor activity

Horizontal locomotor activity of healthy rats was measured in open field plastic cages with dark walls (44 × 44 × 40 cm, without top) (Ugo Basile). Locomotion was recorded by an infrared camera coupled to a computer with AnyMaze Video Tracking System (Stoelting Co. Wood Dale, IL, USA) and was measured as total distance (in cm) travelled during 30 min after s.c. drug administration, analogous to our previous studies^3,15^.

### Defecation

Excreta of individual rats were collected and counted for 1 h after s.c. drug administration in open field plastic cages^3,15^.

### Data handling and statistical analyses

All data were assessed for normal distribution and equal variances by Kolmogorov-Smirnov test and/or D’Agostino and Pearson tests. In dose-response experiments (displacement binding and GTPγS-assay), means of values at each agonist concentration and each pH were calculated and used to derive IC_50_, EC_50_ and the maximum [^35^S]-GTPγS binding by nonlinear regression and were then subjected to unpaired *t*-test for normally distributed data or Mann-Whitney test for non-normally distributed data. To enable direct comparison of the *in vitro* effects of all four compounds, we included some data in Table 1 (calculated pK_a_ of fentanyl, FF3 and NFEPP; experimentally obtained pK_a_ of FF3 and NFEPP, MOR binding of fentanyl, FF3 and NFEPP; EC_50_ of FF3) that were generated in our previous studies^3,15^. Behavioral data were expressed as raw values or transformed to area under the curve (AUC). The net AUC_(4–12 μg/kg)_ values were obtained by calculating the area between the X-axis and the dose-dependency curve of the PPT at 15 min after application of i.v. fentanyl, NFEPP, FF3 or FF6 at doses of 4, 8 and 12 μg/kg. The data presented in Fig. 5 (antinociception and side effects produced by fentanyl, FF3 and NFEPP) were gathered from our previous studies^3,15^, except for FF6 (this study). Notably, the peak effects of fentanyl, FF6 and NFEPP were observed at 10 min^15^ (this study), while those of FF3 were measured at 15 min after injections^3^. To enable direct comparison, we decided to use the effects of all compounds at 15 min after injection. At this time point, fentanyl, FF6 and NFEPP still significantly elevated PPT in inflamed (fentanyl, FF6, NFEPP) and non-inflamed paws (fentanyl, FF6). Transformed data (AUC) were not subjected to statistical analysis. Two-sample comparisons of raw values were made using paired or unpaired *t*-test for normally distributed data, or Wilcoxon or Mann-Whitney test for non-normally distributed data. Changes over time (more than two time points) after one treatment were evaluated using one-way repeated measures (RM) ANOVA followed by Bonferroni test for normally distributed data, or Friedman one-way RM ANOVA followed by Dunn’s test for non-normally distributed data. Two-way RM ANOVA and Bonferroni or Tukey’s test were used to compare two parameters over time. Multiple comparisons at one time point were performed using one-way ANOVA followed by Dunnett’s test or Bonferroni test for normally distributed data, or by Kruskal Wallis one-way ANOVA followed by Dunn’s test for non-normally distributed data. Differences were considered significant if P < 0.05. Prism 5 (GraphPad, San Diego, USA) was used for all tests and graphs and data were expressed as means ± standard error of the mean (SEM) or means ± 95% confidence intervals (Fig. 2B,D and Table 1).
