# Author Correction: pK_a_ of opioid ligands as a discriminating factor for side effects

**DOI:** 10.1038/s41598-020-61224-7

**Published:** 2020-03-04

**Authors:** Giovanna Del Vecchio, Dominika Labuz, Julia Temp, Viola Seitz, Michael Kloner, Roger Negrete, Antonio Rodriguez-Gaztelumendi, Marcus Weber, Halina Machelska, Christoph Stein

**Affiliations:** 1 Charité - Universitätsmedizin Berlin, Campus Benjamin Franklin, Department of Experimental Anesthesiology, Hindenburgdamm 30, 12203 Berlin, Germany; 2 Zuse Institute Berlin, Computational Molecular Design, Takustraße 7, 14195 Berlin, Germany; 3 Present Address: Department of Drug Discovery and In Vitro Pharmacology, Laboratorios Dr. Esteve, Parc Científic de Barcelona, Baldiri Reixac 4-12, Barcelona, Spain

Correction to: *Scientific Reports* 10.1038/s41598-019-55886-1, published online 18 December 2019

This Article contains a typographical error in the figure legend for Figure 5.

“Correlation between pK_a_ values of compounds with antinociception and side effects. (**A,B**) Antinociceptive effects as net AUC_(4–12 μg/kg)_ of PPT (negative values result from subtraction of pre-CFA baseline PPT) at 15 min after i.v. injection of NFEPP, FF3, FF6 and Fen in inflamed (**A**) and contralateral, noninflamed (**B**) paws. AUC values were derived from curves generated by use of n  =  9 (NFEPP and FF3 from^3,15^), n  =  8–10 (FF6, see also Fig. 3) and n  =  19 (Fen) animals. (**C**) Constipation, as assessed by number of fecal boli 1 h after s.c. injection of agonists (30 μg/kg) in relation to the pK_a_ of the substance. Maximum and minimum numbers (with 95% confidence intervals) of boli in controls (vehicle-treated) are shown by the dashed line. Fen (n  =  30) and controls (n  =  34) (always included in all experiments) are averaged across all experiments; NFEPP (n  =  11); FF3 (n  =  12); FF6 (n  =  10). (**D**) Locomotor activity as assessed by the total distance travelled during 30 min after s.c. injection of agonists (30 μg/kg) in relation to the pK_a_ of the substance. Maximum and minimum travelled distances (with 95% CI) in controls (vehicle-treated) are shown by the dashed line. Fen (n  =  31) and controls (n  =  34) (always included in all experiments) are averaged across all experiments; NFEPP (n  =  10); FF3 (n  =  12); FF6 (n  =  10). Graphs show means ± SEM (where available).”

should read:

“Correlation between pK_a_ values of compounds with antinociception and side effects. (**A,B**) Antinociceptive effects as net AUC_(4–12 μg/kg)_ of PPT (negative values result from subtraction of pre-CFA baseline PPT) at 15 min after i.v. injection of NFEPP, FF3, FF6 and Fen in inflamed (**A**) and contralateral, noninflamed (**B**) paws. AUC values were derived from curves generated by use of n  =  9 (NFEPP and FF3 from^3,15^), n  =  8–10 (FF6, see also Fig. 3) and n  =  19 (Fen) animals. (**C**) Constipation, as assessed by number of fecal boli 1 h after s.c. injection of agonists (30 μg/kg) in relation to the pK_a_ of the substance. The dashed line represents vehicle-treated controls (mean of 3 independent experiments). Fen (n  =  30) and controls (n  =  34) (always included in all experiments) are averaged across all experiments; NFEPP (n  =  11); FF3 (n  =  12); FF6 (n  =  10). (**D**) Locomotor activity as assessed by the total distance travelled during 30 min after s.c. injection of agonists (30 μg/kg) in relation to the pK_a_ of the substance. The dashed line represents vehicle-treated controls (mean of 3 independent experiments). Fen (n  =  31) and controls (n  =  34) (always included in all experiments) are averaged across all experiments; NFEPP (n  =  10); FF3 (n  =  12); FF6 (n  =  10). Graphs show means ± SEM (where available).”

Additionally, the Article contains errors in Reference 12 which was incorrectly given as:

Sun J., Chen S. R., Chen H., Pan H. L. mu-Opioid receptors in primary sensory neurons are essential for opioid analgesic effect on acute and inflammatory pain and opioid-induced hyperalgesia. *J Physiol* (2018)

The correct reference is listed below as ref. 1.
